# Rituximab and Pirfenidone in the Treatment of Steroid‐Refractory Bleomycin Lung Injury

**DOI:** 10.1002/rcr2.70228

**Published:** 2025-07-14

**Authors:** Aasir M. Suliman, Irfan Ul Haq, Khalid Albsheer, Mohamed Elgara

**Affiliations:** ^1^ Pulmonology Department Hamad General Hospital, Hamad Medical Corporation Doha Qatar

**Keywords:** bleomycin, lung injury, pirfenidone, pneumonitis, rituximab

## Abstract

Bleomycin‐induced lung injury (BILI) is a well‐recognised but potentially life‐threatening complication of ABVD chemotherapy (adriamycin, bleomycin, vinblastine and dacarbazine), often requiring prompt diagnosis and intervention. We present a case of a 43‐year‐old female with Hodgkin's lymphoma who developed progressive respiratory symptoms following her fourth cycle of ABVD. A broad infectious and autoimmune workup was unremarkable, and chest imaging was consistent with interstitial lung disease. Her condition deteriorated despite empirical antibiotics and high‐dose corticosteroids. The addition of rituximab and pirfenidone led to significant clinical and radiological improvement, highlighting the potential role of these agents in managing steroid‐refractory BILI.

## Graphical abstract

Bleomycin‐induced lung injury (BILI) is a serious, potentially life‐threatening complication of ABVD chemotherapy. Diagnosis is often challenging and made by exclusion. Treatment becomes particularly difficult when corticosteroids fail, as alternative options are limited. This case demonstrates a favourable response to rituximab and pirfenidone, suggesting a potential alternative therapeutic approach.

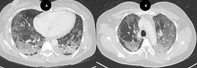

## 1 Introduction

Bleomycin, a chemotherapeutic agent commonly used in the ABVD regimen (adriamycin, bleomycin, vinblastine and dacarbazine) for Hodgkin's lymphoma, is associated with pulmonary toxicity in up to 18% of patients, with fetal forms occurring in approximately 4% [1]. The clinical presentation of bleomycin‐induced lung injury (BILI) is variable and may mimic other lung diseases, often posing a diagnostic dilemma [2]. Prompt recognition and withdrawal of bleomycin are critical. While corticosteroids are widely considered the standard treatment for symptomatic patients, responses to steroid therapy alone can be suboptimal [3]. Here, we present a rare case of severe BILI that responded favourably to rituximab and pirfenidone after steroid failure.

## 2 Case Report

**FIGURE 1:**
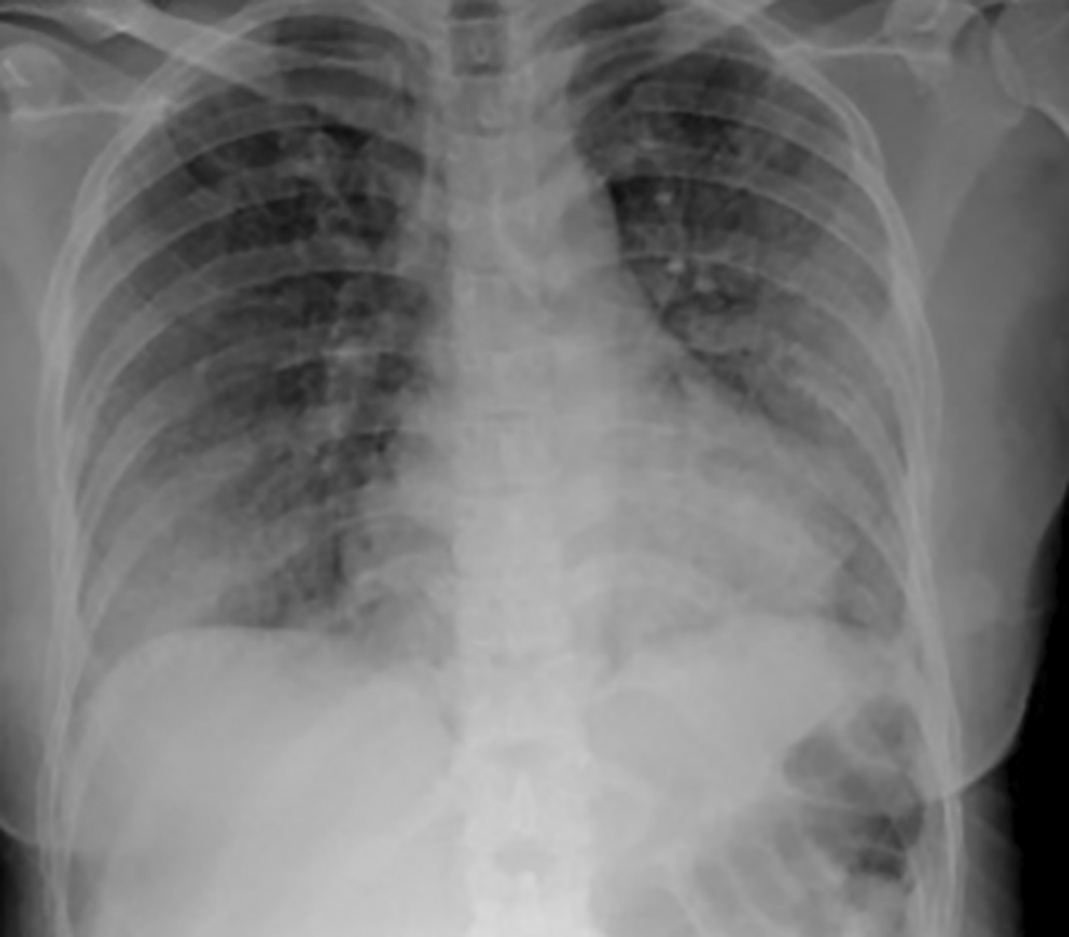
Chest X‐ray demonstrates poorly defined bilateral airspace opacities.

A 43‐year‐old Asian female with stage III/IV Hodgkin's lymphoma achieved remission after three cycles of ABVD chemotherapy. Four weeks following her fourth cycle, she presented with a 10‐day history of a progressive dry cough, shortness of breath, and fever, without preceding flu‐like symptoms or other systemic complaints. Upon admission, she was febrile (38.5°C), tachypneic (respiratory rate 28/min) and hypoxic (SpO_2_ 88% on room air). Initial laboratory tests revealed leukocytosis (WBC 14.2 × 10^3^/μL) and an elevated CRP (120 mg/L). Other tests, including renal and liver functions, were within normal limits. Chest X‐ray demonstrated bilateral diffuse infiltrates (Figure 1), while echocardiography ruled out cardiac involvement. Suspecting atypical pneumonia, she was started on broad‐spectrum antibiotics (ceftriaxone with azithromycin).

**FIGURE 2:**
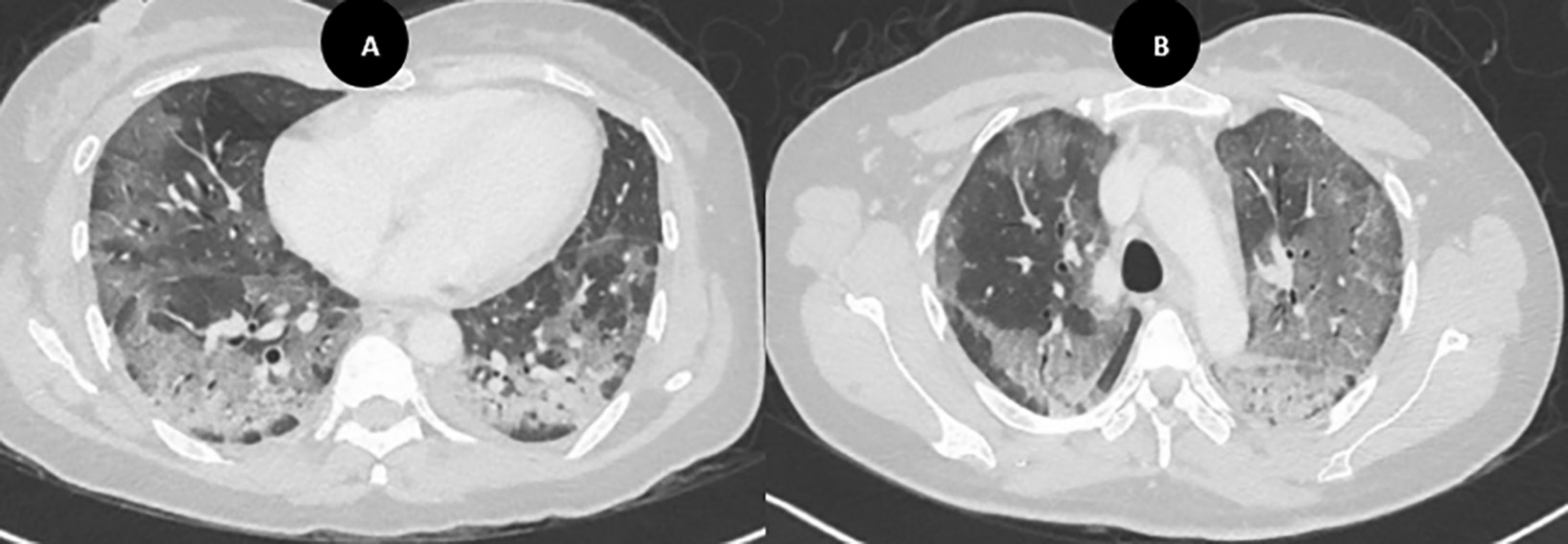
(A, B) Initial HRCT performed on day 3 of admission shows bilateral, diffuse, patchy ground‐glass opacities with subtle interlobular septal thickening.

Despite treatment, her respiratory status deteriorated over the following 72 h, necessitating 60% FiO_2_ via a Venturi mask. A high‐resolution computed tomography (HRCT) scan of the chest revealed widespread bilateral ground‐glass opacities with interlobular septal thickening (Figure 2). To rule out atypical and opportunistic infection, particularly Pneumocystis jirovecii pneumonia (PCP), bronchoscopy with bronchoalveolar lavage (BAL) and washings was performed. The procedure revealed normal bronchial anatomy, and microbiological and cytological studies were negative. An extended connective tissue disease panel, including anti‐MDA‐5 antibodies, also returned negative. PET‐CT demonstrated heterogeneous pulmonary uptake corresponding to the interstitial opacities but showed no evidence of malignancy. Notably, pre‐chemotherapy spirometry and diffusing capacity of the lungs for carbon monoxide (DLCO) had been within normal limits.

**FIGURE 3:**
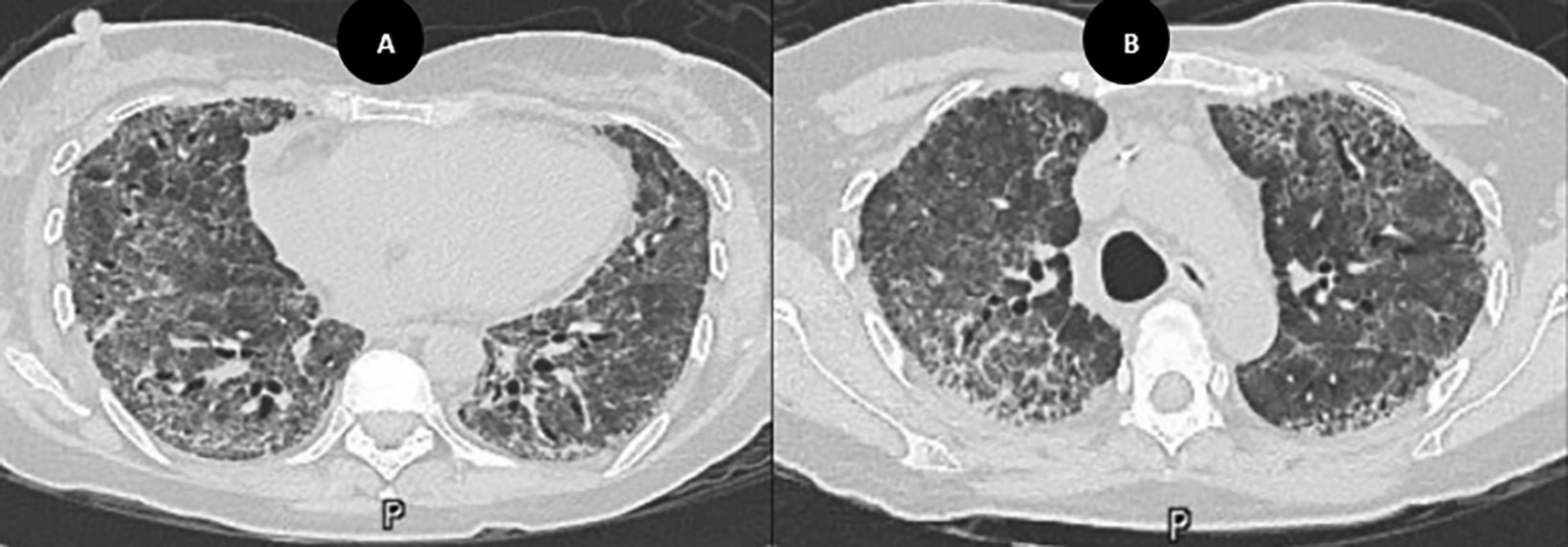
(A, B) Follow‐up HRCT performed 4 weeks after admission demonstrates interval improvement in the widespread ground‐glass opacities, along with evolving fibrotic changes, including interlobular septal thickening and traction bronchiectasis.

Given the non‐diagnostic workup, BILI was considered the leading diagnosis. High‐dose intravenous methylprednisolone (1000 mg daily) was administered for 3 days but failed to improve her hypoxemia. She was subsequently transitioned to a tapering regimen of oral corticosteroids. Although a surgical lung biopsy was offered, the patient declined. Following a multidisciplinary discussion, intravenous rituximab (1000 mg) was initiated as salvage therapy. Remarkably, her oxygen requirements decreased significantly within 7 days, allowing for transition to a nasal cannula at 3 L/min. Two weeks later, at the time of the planned second rituximab dose, a follow‐up chest CT showed regression of ground‐glass opacities but progression of fibrotic changes (Figure 3). As a result, the second rituximab dose was withheld, and pirfenidone was initiated as an anti‐fibrotic agent alongside continued steroid tapering.

**FIGURE 4:**
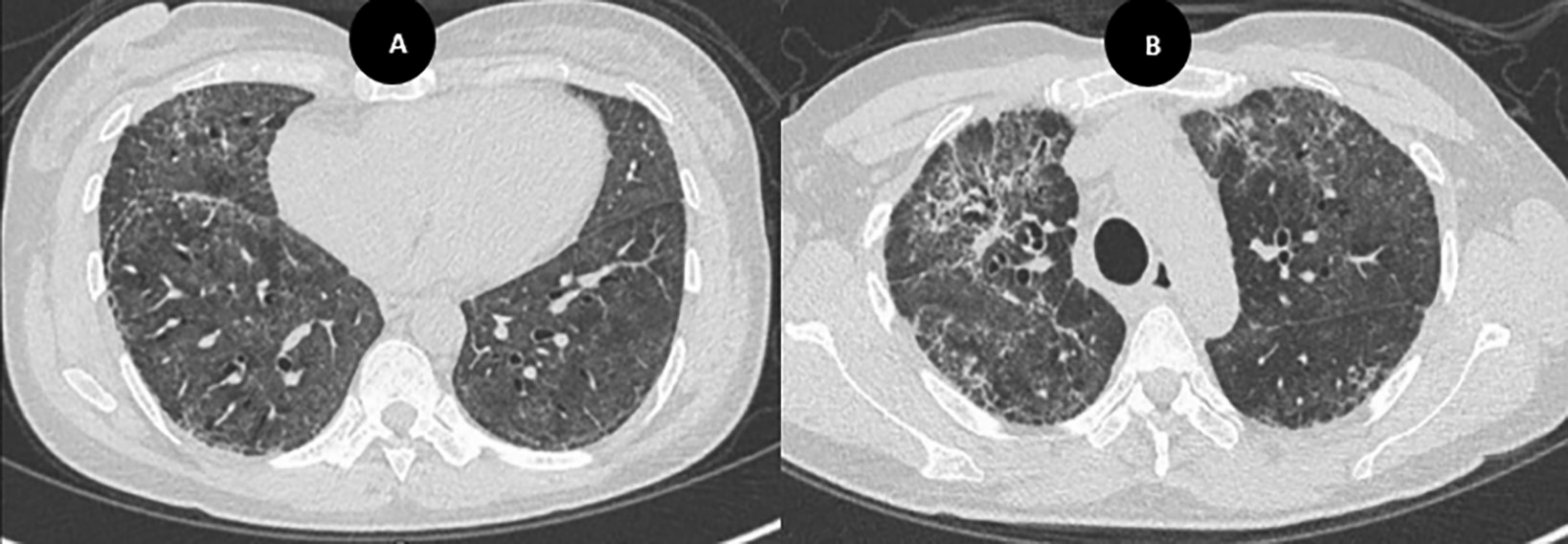
(A, B) HRCT performed 3 months post‐discharge shows continued resolution of ground‐glass opacities, with overall improvement in fibrotic changes, except for a persistent focal area in the right upper lobe.

After approximately 6 weeks of hospitalisation, the patient was discharged home on 1 L/min of supplemental oxygen. She enrolled in outpatient pulmonary rehabilitation and demonstrated sustained clinical improvement. At her 3‐month follow‐up, she required only minimal oxygen support (1 L/min via nasal cannula) during exertion, and repeat imaging showed significant radiological improvement (Figure 4). Steroid tapering was continued, alongside ongoing treatment with pirfenidone.

## 3 Discussion

BILI is a well‐documented complication of ABVD chemotherapy, characterised by pneumonitis and pulmonary fibrosis [4]. The pathogenesis of BILI is not fully understood, but it is thought to involve massive oxidative stress, alveolar epithelial cell death, fibroblast proliferation, and infiltration of immune cells into the lung tissue, leading to inflammation and fibrosis. The risk is increased with high cumulative doses or impaired drug clearance and is further modulated by factors such as age, renal impairment, prior chest radiation and supplemental oxygen use [5].

BILI lacks distinct clinical, radiological or pathological features; therefore, its diagnosis relies on excluding other potential causes of lung involvement and identifying radiologic and functional findings consistent with BILI. Characteristic findings include a reduced diffusion capacity on pulmonary function tests and bilateral opacities on HRCT [6].

Although corticosteroids are typically first‐line therapy for BILI, a significant subset of patients may show minimal or no improvement which necessitates establishing other alternative therapies [3]. A limited number of case studies have examined the use of imatinib in BILI with variable effects on outcomes [7]. Similarly, tumour necrosis factor‐alpha (TNF‐α) inhibitors such as infliximab have shown limited efficacy when used in advanced stages of the disease [8].

In this case, the addition of Rituximab, a monoclonal antibody targeting CD20 on B‐cells, was considered a salvage immunomodulatory agent based on emerging evidence suggesting its role in steroid‐refractory inflammatory interstitial lung diseases [9]. The rationale for using this molecule is that it allows a systemic deactivation of the immune system to prevent the progression of fibrosis, stabilising and possibly improving the condition of patients with pulmonary disease from a clinical, functional and radiological point of view [10]. The observed improvement in our patient following rituximab may support its utility in this context.

Bleomycin is commonly used in animal models to simulate idiopathic pulmonary fibrosis (IPF) for research purposes, given the similarity in their pathogenic mechanisms [11]. This shared pathogenesis has led to the hypothesis that treatments effective in IPF may also be beneficial in managing BILI. Supporting this notion, several reports have documented favourable outcomes with the use of anti‐fibrotic agents such as pirfenidone and nintedanib in BILI [6, 12]. In our case, pirfenidone, an anti‐fibrotic agent known to inhibit transforming growth factor‐beta (TGF‐β), a key mediator of fibrosis, was introduced in response to the fibrotic changes noted on imaging. Its addition may have played a role in limiting fibrotic progression and facilitating further clinical recovery.

BILI is a severe complication of chemotherapy that can be challenging to manage, particularly in steroid‐refractory cases. This case highlights the potential therapeutic benefit of rituximab and pirfenidone to target both the inflammatory and fibrotic components of the disease. Early recognition and a multidisciplinary approach are critical in optimising outcomes for these patients, and further research is needed to establish the efficacy and safety of these agents in the treatment of BILI. It is inappropriate to draw any conclusions about the effectiveness of treatment based on only one case, so further accumulation of data on treatment is desirable.

## Author Contributions

A.M.S. conceived and designed the idea, conducted the literature review, wrote the manuscript, supervised the project and proof‐read the manuscript. I.U.H. critically revised the paper and helped in manuscript writing. K.A. contributed to the literature review and drafted the initial manuscript. M.E. contributed to the literature review and drafted the initial manuscript. All authors gave final approval of the version to be published and agreed to be accountable for all aspects of the work.

## Ethics Statement

The case report is approved by the Medical Research Center at Hamad Medical Corporation and the Hamad Institutional Review Board (IRB) under number MRC‐04‐25‐542.

## Consent

This case report does not contain any personal identifier of the patient (such as name, photograph… etc.). It only includes radiological imaging, which does not contain any identifications. Written informed consent was obtained from the patient for publication of this case report and any accompanying images.

## Conflicts of Interest

The authors declare no conflicts of interest.

## Data Availability

The data sets used and/or analysed during the current study are available from the corresponding author upon reasonable request.
